# Congenital cutis laxa

**DOI:** 10.4103/0256-4947.60528

**Published:** 2010

**Authors:** Anupama Mauskar, Preeti Shanbag, Varsha Ahirrao, Leena Nagotkar

**Affiliations:** Department of Pediatrics, Lokmanya Tilak Municipal Medical College and General Hospital. Sion, Mumbai - 400 022, Maharashtra, India

**To the Editor:** Cutis laxa (CL) is a group of inherited and acquired disorders characterized by loose and redundant skin with reduced elasticity.^1^ Autosomal dominant, autosomal recessive, and X-linked recessive patterns have been noted in the inherited forms.^2^–^4^ The autosomal dominant form has a benign course; primarily, skin involvement is present, with few, if any, systemic complications, and a normal life expectancy. It can be caused by mutations in the elastin gene, but molecular heterogeneity cannot be excluded.^2^,^3^ Type I autosomal recessive CL is characterized by pulmonary emphysema, umbilical and inguinal hernias, and gastrointestinal and vesico-urinary tract diverticula, and has the poorest prognosis. The Type II recessive form is called CL and is associated with joint laxity and developmental delay.^4^ The histopathology of the skin in patients with CL reveals loss and/or fragmentation of elastic fibers.^5^,^6^ All forms are very rare and no precise data about their prevalence are available.

**Figure 1:**
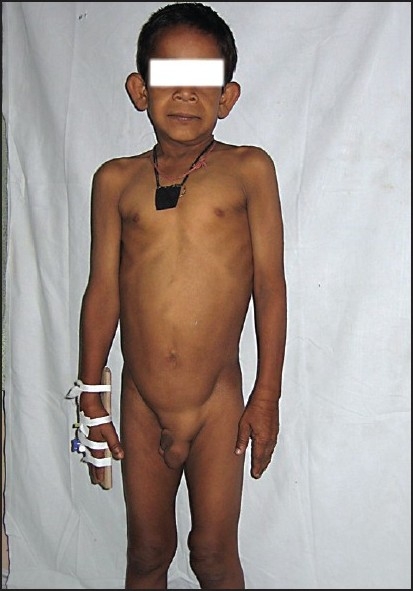
Senile appearance of the face, everted nostril, wrinkling over the dorsum of the hands, left-sided inguinal hernia, and small paraumbilical hernia.

**Figure 2:**
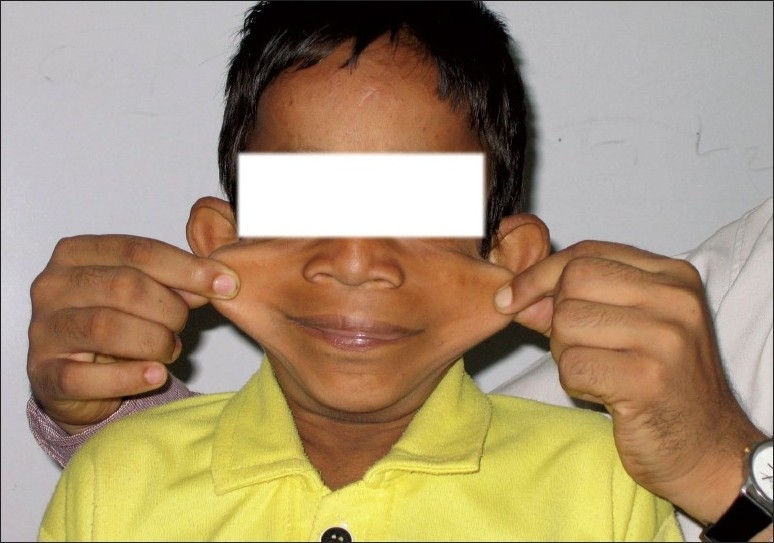
Laxity of skin.

We report an eight-year-old boy with congenital CL with umbilical and paraumbilical hernias, emphysema, and pulmonary artery branch stenosis. The child presented with fever and cough of seven days duration and four days of breathlessness of days duration. He was receiving treatment from a private practitioner, but was referred for increasing breathlessness. There was a history of recurrent episodes of cough and breathlessness since infancy, with an increase in the frequency and severity of the episodes in the past year. The patient had responded to oral medications in earlier episodes. This was the third child of nonconsanguineous parents with no similar family history. Early development was normal and the child was currently in the third grade and school performance was average. Increased laxity of the skin had been noticed six months of age, but a dermatology opinion was not sought. There was no history of skin rash or reaction to any drugs in the past. On examination, the child was febrile and had respiratory distress with intercostal and subcostal retractions. The respiratory rate was 88/min, heart rate 128/min, and blood pressure 100/70 mm Hg. There was no cyanosis or clubbing. The face had a senile appearance with an antimongoloid slant and slightly everted nostrils. The skin was loose and hanging in folds over the dorsum of the trunk and wrinkled over the face and on the dorsum of the hands. There was a left-sided, reducible, inguinal hernia and a small paraumbilical hernia (Figures 1 and 2) there was no laxity of joints. The weight and height were 20.5 kg and 118.5 cm, respectively, both at the fifth percentile for that age. Chest examination revealed an increased anteroposterior diameter with a Harrison sulcus. Rhonchi and coarse crepitations were heard bilaterally. There was no cardiomegaly; first and second heart sounds were heard normally. A Grade II ejection systolic murmur was heard in the pulmonary area. Examination of the abdomen revealed a palpable liver, 2 cm below the right costal margin, and a just-palpable spleen. Other systems were normal.

**Figure 3:**
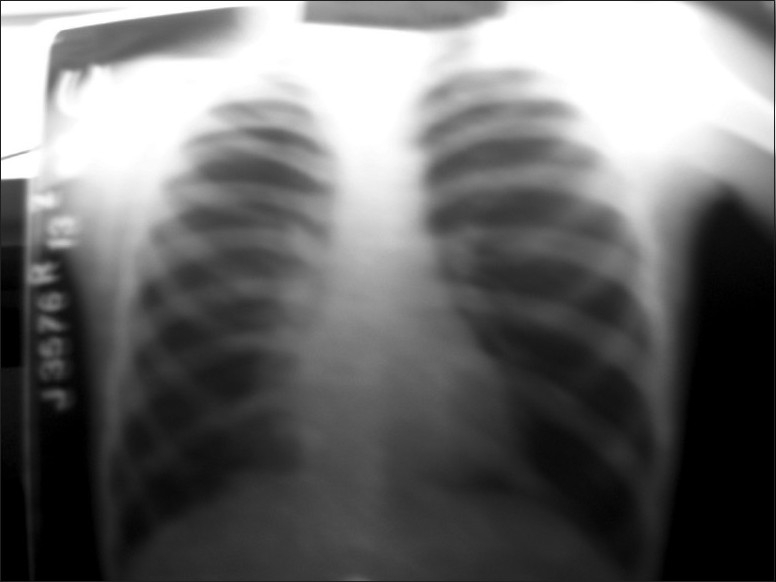
Chest x-ray showing emphysema.

Tests showed a hemoglobin of 12 g/dL, a total WBC count of 7800/mm^3^ with a differential count of polymorphs of 78%, lymphocytes of 20%, and eosinophils of 2%. A chest x-ray showed emphysema with patchy opacities in the right lower zone (Figure 3). Arterial blood gases showed no hypoxemia. There was no right ventricular hypertrophy on electrocardiography. Two-dimensional echocardiography showed stenosis of both the right and left pulmonary arteries. The child was treated with bronchodilators and antibiotics and was discharged after seven days. However, he was readmitted two weeks later for repair of his left inguinal hernia. Postoperative recovery was uneventful.

The diagnosis of a CL syndrome is based on clinical assessment of the typical skin features and the associated extracutaneous findings. In our patient, there was no history of any similar problem in any of the family members, thus ruling out an autosomal dominant inheritance. He had no history of developmental delay nor did he have any joint laxity, as described for Type II recessive CL; our patient probably suffered from the Type I recessive form. He had the characteristic cutaneous abnormalities described in all the varieties of CL. Additionally, he had paraumbilical and inguinal hernias, pulmonary emphysema, and stenosis of the right and left pulmonary arteries. Cardiopulmonary abnormalities are common in Type I recessive CL and are the main factors to determine the prognosis and life expectancy. Pulmonary emphysema, cor pulmonale, and right-sided heart failure caused by pulmonary disease have been commonly described. Various cardiovascular abnormalities including aortic aneurysm, pulmonary artery multiple branch stenosis, as in our patient, and pulmonary valve stenosis have been reported with this form of congenital CL.^1^,^7^,^8^
